# Supplementary figures and images for: Translational profiling of macrophages infected with Leishmania donovani identifies mTOR- and eIF4A-sensitive immune-related transcripts
Source: PLoS Pathog. 2020 Jun 1;16(6):e1008291. doi: 10.1371/journal.ppat.1008291 (PMC7310862; doi:10.1371/journal.ppat.1008291)

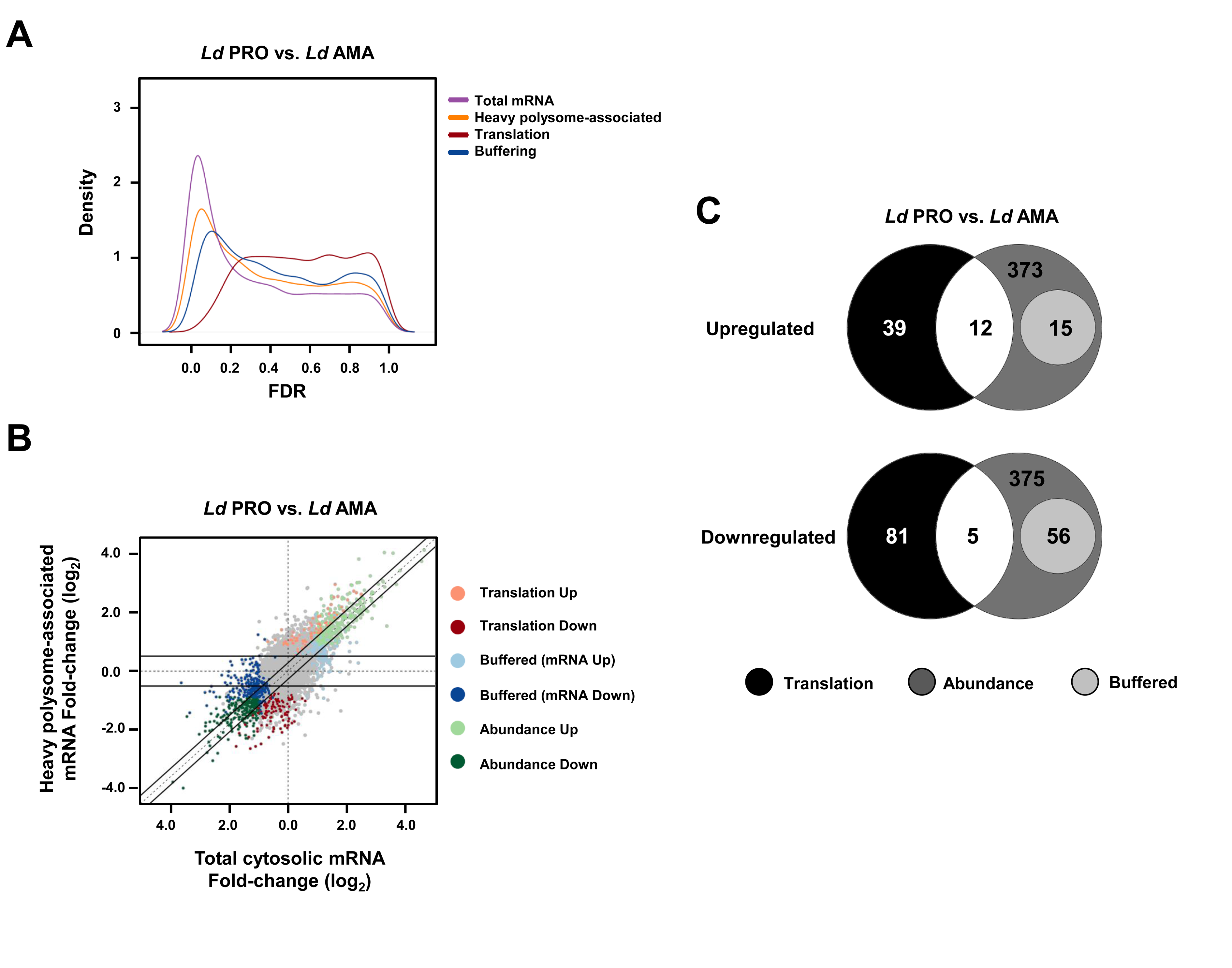

Supplement: S1 Fig — (A) Kernel densities of adjusted p values (FDRs) following anota2seq analysis on changes in total mRNA, heavy polysome-associated mRNA, translational efficiency, and translational buffering comparing Ld PRO to Ld AMA-infected BMDMs (n ≥ 3). (B) Scatter plot of log2 fold changes (for the same comparisons as in panel A) for heavy polysome-associated mRNA and total cytosolic mRNA. Differentially regulated transcripts through translation, abundance or buffering are indicated. Unchanged mRNAs are shown in grey (n ≥ 3) (C) Venn diagrams indicating the number of mRNAs up- or down-regulated at the level of translation, abundance, and buffering for Ld PRO-infected BMDMs compared to Ld AMA-infected BMDMs. (TIF) [file ppat.1008291.s001.tif]

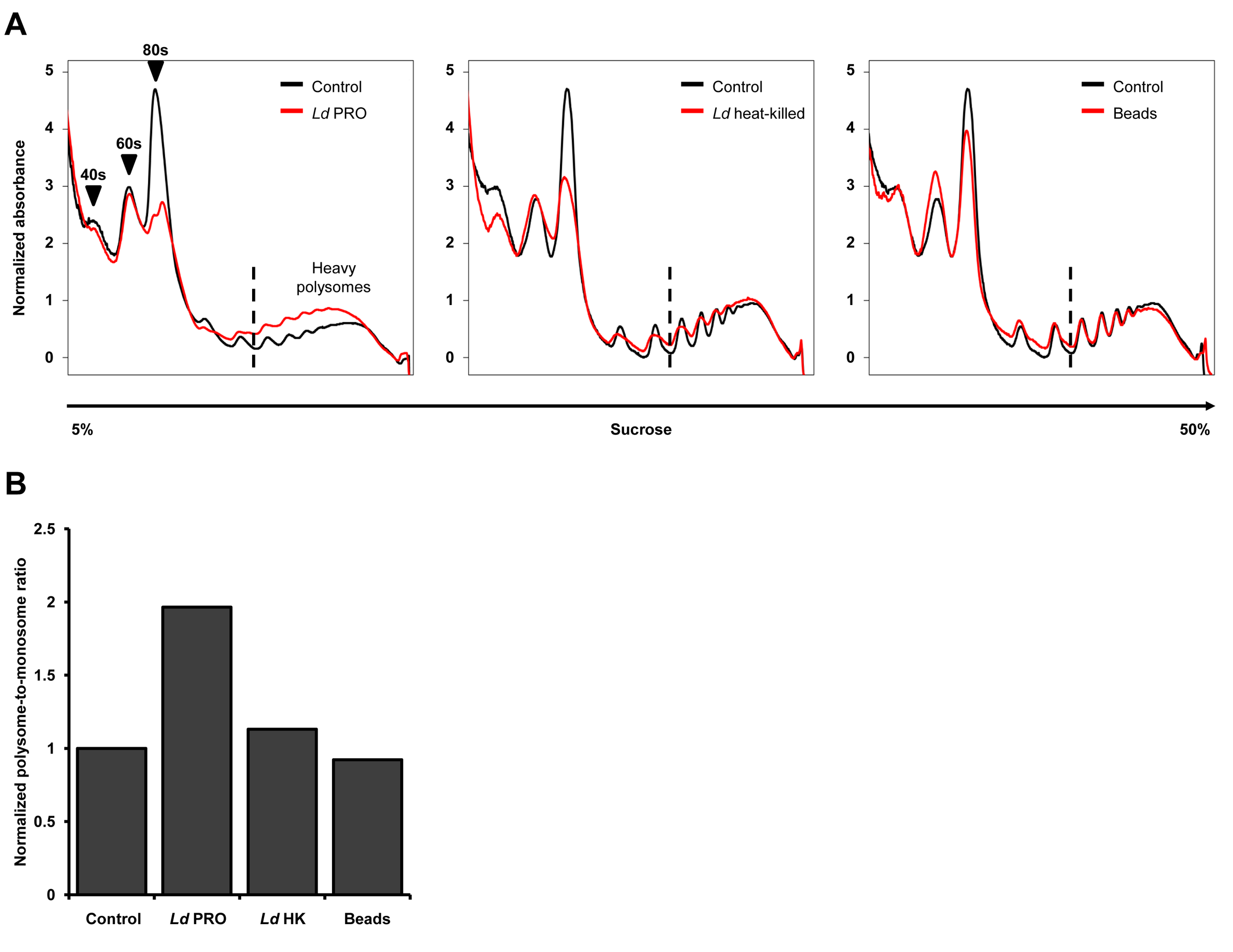

Supplement: S2 Fig — (A) BMDM cultures were inoculated with 10:1 live L. donovani promastigotes (Ld PRO) or heat-killed Ld PRO (Ld HK), latex beads, or left untreated (control) for 6 h. Cell lysates were sedimented on 5 to 50% sucrose gradients. Gradients were fractionated, and the absorbance at 254 nm was recorded continuously. Absorbance values were normalized. Arrows indicate the 40S and 60S ribosomal subunits and 80S (monosomes). The heavy polysome regions were identified as fractions containing mRNAs associated with >3 ribosomes (i.e. efficiently translated mRNAs). (B) The area under the curve of the monosome and heavy polysome regions was calculated, and the heavy polysome-to-monosome ratios were then normalized to values for control BMDM cultures. Data are representative of three independent experiments. (TIF) [file ppat.1008291.s002.tif]

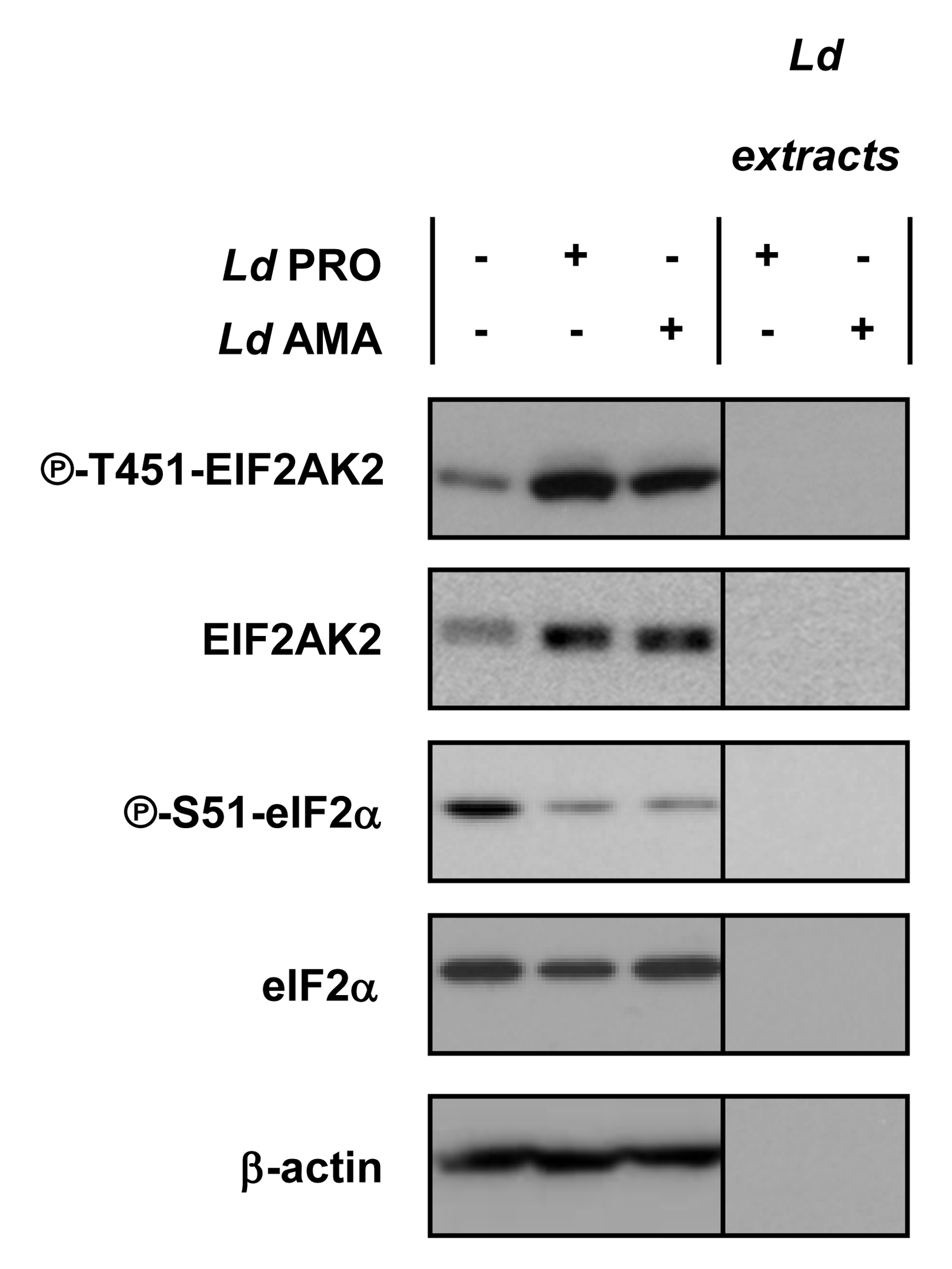

Supplement: S3 Fig — BMDM cultures were inoculated with Ld AMA, Ld PRO or left uninfected for 6 h. Phosphorylation and expression levels of EIF2AK2 and eIF2α were monitored by Western blotting. Total amounts of β-actin were used as a loading control. Total protein extracts from Ld cultures were used to control for any cross-reactivity of the antibodies against parasite antigens. Data are representative of three independent experiments. (TIF) [file ppat.1008291.s003.tif]

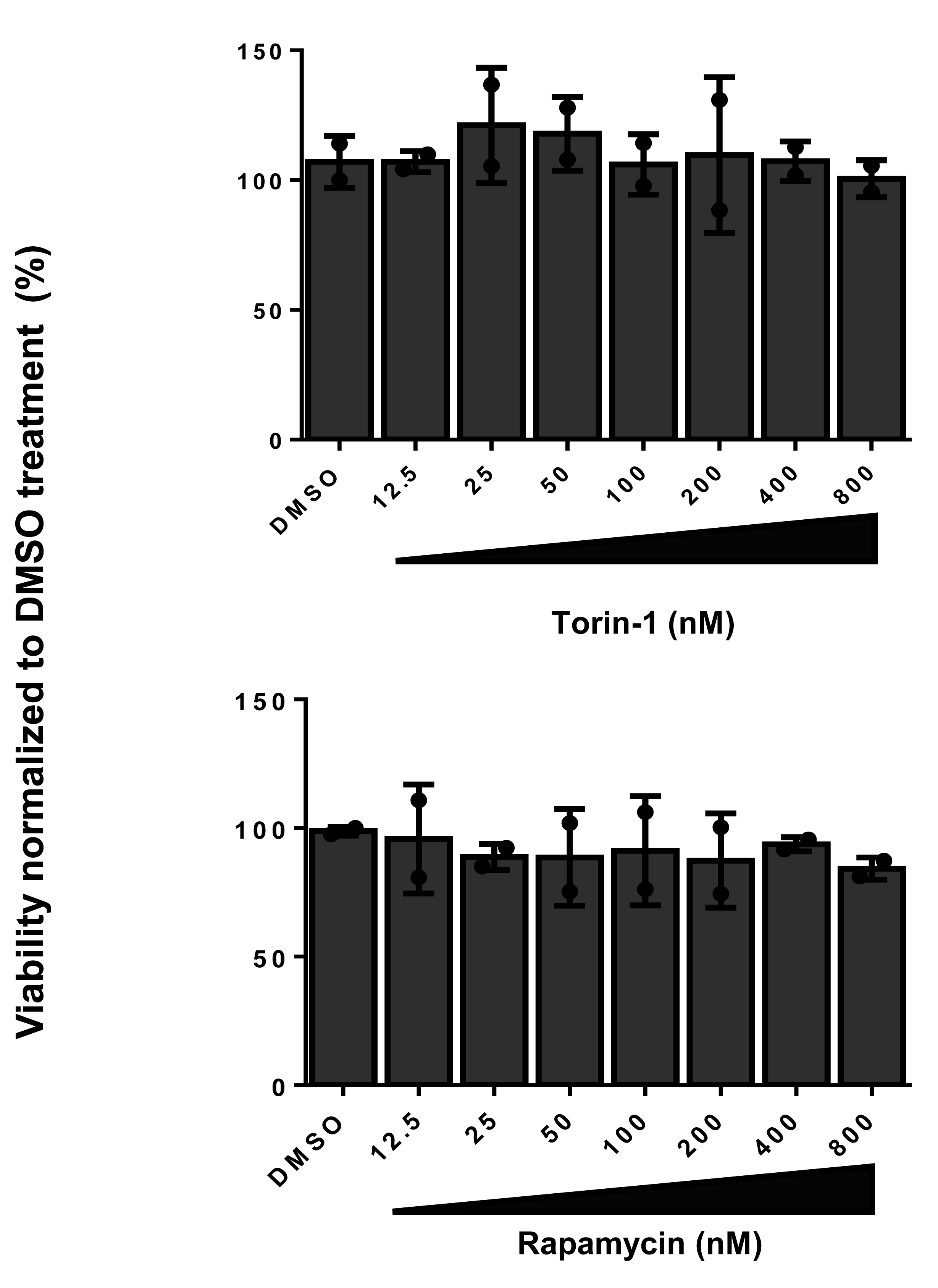

Supplement: S4 Fig — L. donovani cultures were treated with increasing concentrations of rapamycin (2.5–160 nM), Torin-1 (12.5–800 nM) or an equivalent volume of DMSO (vehicle) for 24 h. Acute toxicity of the inhibitors was measured by resazurin assays. Percent viability was normalized to DMSO-treated parasites. Data are representative of two independent experiments performed in technical triplicates. (TIF) [file ppat.1008291.s004.tif]

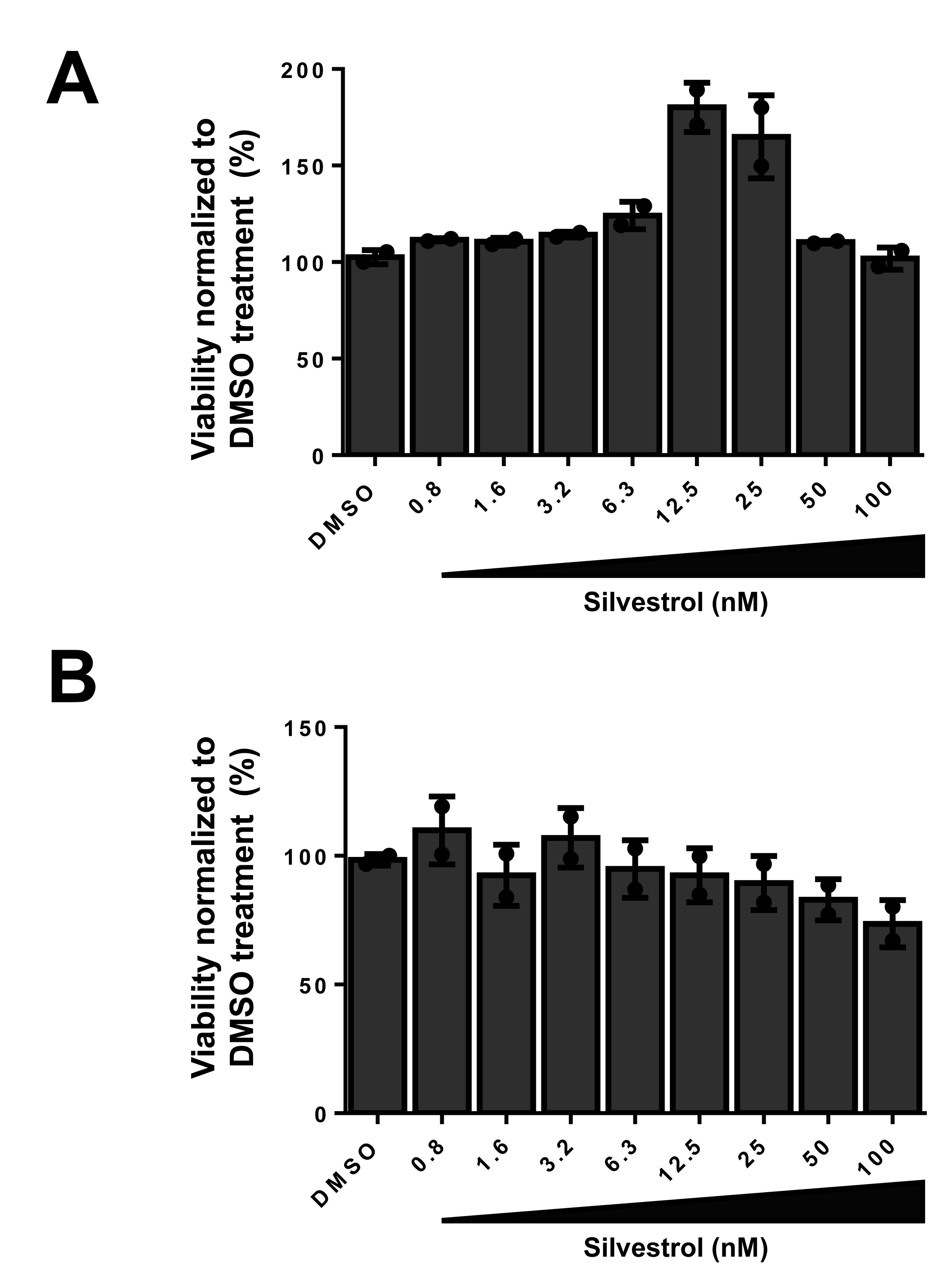

Supplement: S5 Fig — (A) BMDM and (B) L. donovani cultures were treated with increasing concentrations of silvestrol (0.8–100 nM) or an equivalent volume of DMSO (vehicle) for 24 h. Acute toxicity of the inhibitor was measured by resazurin assays. Percent viability was normalized to DMSO-treated parasites. Data are representative of two independent experiments performed in technical triplicates. (TIF) [file ppat.1008291.s005.tif]

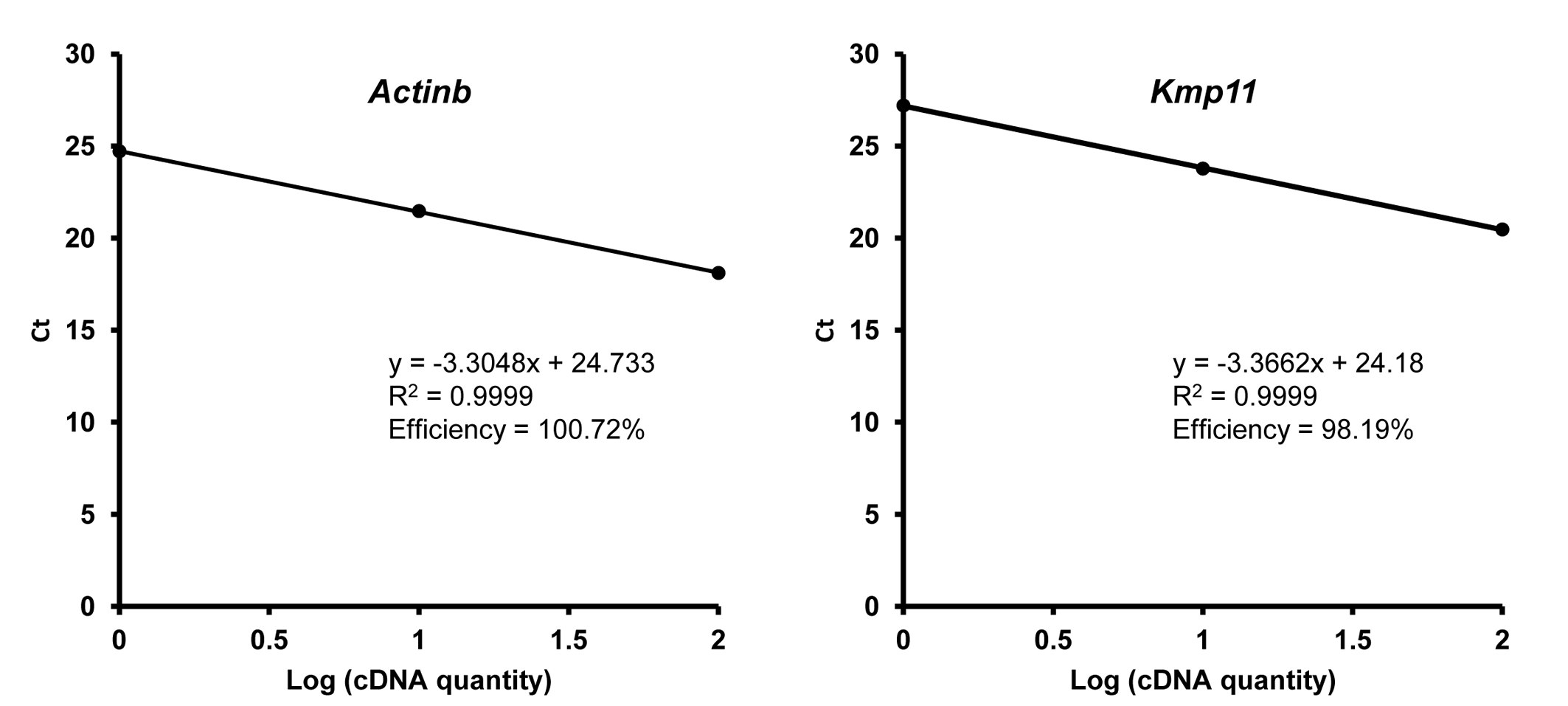

Supplement: S6 Fig — The efficiency of the primers used to amplify mouse Actinb and Leishmania Kmp11 genes was verified by measuring Ct values along a serial dilution of cDNA (plotted as Log[cDNA amount]). Efficiency was calculated using ThermoFisher's online qPCR Efficiency Calculator software. (TIF) [file ppat.1008291.s006.tif]
