# Supplementary material for: Translational profiling of macrophages infected with Leishmania donovani identifies mTOR- and eIF4A-sensitive immune-related transcripts
Source: PLoS Pathog. 2020 Jun 1;16(6):e1008291. doi: 10.1371/journal.ppat.1008291 (PMC7310862; doi:10.1371/journal.ppat.1008291)
Supplement: S4 Table — (DOCX) [file ppat.1008291.s010.docx]

**Table S4. Primer sequences used for RT-qPCR analyses**

| **Gene** |  | **Primer sequence (5' - 3')** |
| --- | --- | --- |
| Mouse *Actb* | Forward | 5'- CACCCACACTGTGCCCATCTACGA -3' |
|  | Reverse | 5'- CAGCGGAACCGCTCATTGCCAATGG -3' |
| Leishmania *Kmp11* | Forward | 5'- GCCTGGATGAGGAGTTCAACA -3' |
|  | Reverse | 5'- GTGCTCCTTCATCTCGGG -3' |
| Mouse *Pabcp1* | Forward | 5'- CGCAAATTTGAGCAGATGAAGC -3' |
|  | Reverse | 5'- CCGGAGACGCTCATCATCAATC -3' |
| Mouse *Eif2ak2* | Forward | 5'- CGTTTCTTGCCTCCTGCTTTG -3' |
|  | Reverse | 5'- GGGACCTCCACATGACAGAAG -3' |
| Mouse *Tgfb1* | Forward | 5'- AGCCCGAAGCGGACTACTATG -3' |
|  | Reverse | 5'- TAATCTCTGCAAGCGCAGCTC -3' |
